# Knowledge, attitude and practice related to diabetes mellitus among the general public in Galle district in Southern Sri Lanka: a pilot study

**DOI:** 10.1186/s12889-017-4459-5

**Published:** 2017-06-01

**Authors:** H. M. M Herath, N. P. Weerasinghe, H. Dias, T. P. Weerarathna

**Affiliations:** 1 Department of Medicine, Faculty of Medicine, University of Ruhuna, P.O. Box 70, Galle, Sri Lanka; 2 Department of Microbiology, Faculty of Medicine, University of Ruhuna, P.O. Box 70, Galle, Sri Lanka

**Keywords:** Sri Lanka, Diabetes mellitus, South Asians, General public, Knowledge, Attitude, Practices

## Abstract

**Background:**

Type 2 diabetes mellitus (DM) has become a global epidemic with significant disability and premature death. Identification of the level of knowledge, attitude and practice (KAP) related to diabetes among the general public is important in strategies for prevention of diabetes mellitus.

**Methods:**

This study was conducted as a community based cross sectional study in three Medical Officers of Health (MOH) areas in Galle district. Previously healthy literate individuals who have not attended any diabetes education program in the last two years were selected for this study.

**Results:**

A total of 277 participants were included in the study. The majority (77%) had either moderate (39%) or above moderate knowledge (38%) on diabetes mellitus. Even though, level of education was significantly and positively associated with knowledge (*p* = 0.001), the association of gender and age with knowledge was not significant. Unlike knowledge, the attitude towards diabetes was poor in majority (90%) and level of education had no significant effect on attitude. With regards to practices, more than half of study subjects never had their blood sugar checked and, about 65% used to take refined sugar liberally and a large majority (80%) had no regular exercise activity.

**Conclusions:**

Even though the majority (77%) had moderate or above moderate knowledge on diabetes, their attitudes towards diabetes was poor (88%). It appears that the higher knowledge on diabetes did not translate into good practices as over 50% of study subjects did not involve with any preventive measures. Therefore, more emphasis should be given to address the issue of poor attitude and practices towards diabetes mellitus among general public in Sri Lanka.

## Background

Type 2 diabetes mellitus (DM) has become a global epidemic with significant disability, premature death and enormous medical costs [1]. Total number of people with diabetes is projected to double between 2000 and 2030 with a significantly greater rise in Asia [1]. Among Asian regions, South Asia is developing as the epicentre of this escalating epidemic, reflecting rapid transitions in demography, unhealthy diet and lifestyle patterns [2]. People living in the South Asia are also at higher risk for developing DM at relatively younger age and at a lower body mass index than other ethnic groups [3]. As a result, there has been a dramatic rise in the number of patients with DM in South Asia, which in turn places urgent demands on health care systems in these countries, most of which are ill-prepared for such demands.

Problems associated with DM can be minimized by early diagnosis and proper management [4]. The primary aim of management of DM is to delay the macro and microvascular complications by achieving optimal glycaemic control [4]. This involves lifestyle modification, including regular exercise, healthy diet and weight loss, and drug therapy. Therefore, health literacy is an integral part of the diabetes management. Patients with good knowledge on diabetes and its complications seek proper treatment and care, and take charge of their health [5]. There is strong evidence that individuals who are educated and diligent with their diabetes self-care achieve better and durable diabetic control [6, 7]. Furthermore, previous studies on knowledge, attitude and practice (KAP) on diabetes have supported the needs of greater awareness of prevention, diagnosis, and risk factor control in diabetes [8].

Even though, having better knowledge, good attitude and practices on diabetes could be helpful for better management, there is a paucity of evidence of current knowledge, attitude and practice related to diabetes among the general public in Sri Lanka. A previous clinic based study in Sri Lanka had revealed a significant knowledge gap among diagnosed patients with DM [9]. However, there are no previous studies conducted in Sri Lanka to assess the KAP of the general public related diabetes and its complications [9]. Identification of knowledge, attitudes and practices related to diabetes mellitus in the general public would provide better insight for the development of preventive strategies specific to the Sri Lankan context. Therefore, this study was conducted to evaluate knowledge, attitude and practice towards diabetes in a cohort of general public in Sri Lanka.

## Methods

### Study setting

This study was conducted as a community based cross sectional study from January to December 2015 in three Medical Officer of Heath (MOH) areas (Galle, Akmeemana and Bope-Poddala) of Galle district of Southern Sri Lanka. These three MOH areas have an estimated population of 140,000.

### Study population

The study population was previously healthy adults aged 18 years and above from both genders. People who were less than 18 years old, mentally handicapped, unable to read and write in Sinhala or Tamil languages, had attended a diabetes education program in the last two years,or declined to participate in this study were excluded.

### Study design and procedures

Cluster sampling technique was used to select households for this study. Based on the building density map of three MOH areas, 17 clusters were selected to cover all areas of population density. From each cluster, approximately 20 households were randomly selected first by identifying a random location based on voters’ list of the area and then by selecting eligible households from the selected location, until meeting the required number of households per cluster, according to the World Health Organization EPI Methodology. One individual from each household was then chosen for the study by open invitation method. Thus, a total of 277 previously healthy individuals were selected. They were invited to the Faculty of Medicine, Galle for data collection. Information on KAP on diabetes and other socio-demographic factors were collected. Data were collected using a pre-designed questionnaire by two trained medical officers.

### Development of KAP questionnaire and data collection

Questionnaire used in this study was developed to collect data on participants’ KAP regarding diabetes, its risk factors and management. Main domains related to KAP were recognised after extensive review of similar, but validated questionnaires used in other settings [10–13]. The necessary modifications were done considering lifestyle, social, cultural and economic factors related to Sri Lankan population [14]. Face validity of the questionnaire was ensured independently by two experts dealing with diabetic patients. Subsequent to this, the content validity of the questionnaire was done after number of meetings with several other experts in diabetes (two physicians, a dietician, a diabetes educator, a nurse, a psychologist and three patients with diabetes. Finally, the questionnaire was administered to five randomly selected individuals with no history of DM to improve the clarity of questions. The final version of the questionnaire was used for this study.

The first part of the questionnaire covered the respondent’s demographic information which included: age, sex, race, religion, level of education, occupation and average monthly income. Knowledge was measured using 8 main questions related to diagnosis, risk factors, prevention, and complications of DM. Examples of questions covering knowledge were “What happens to blood sugar in diabetes?”, “Dysfunction of which of the following organs leads to DM?”, “What is the best way to diagnose DM?”. “Do you think diabetes can affect other organs?”.Answers were provided with three categorical responses “yes”, “No” and “Don’t know” followed by correct and incorrect responses to further evaluate the responses. One point was offered for each correct response and the total score was calculated. Score ranges of 0–13, 14–18 and 19–26 were considered as poor, moderate and good knowledge respectively.

An attitude was assessed using seven questions related to adherence to treatment of DM. The questions were,

- “Do you think that controlling glucose with diet alone is superior to that of controlling glucose with diet and medications?”
- “Can long term use of metformin cause kidney damage?”
- “Does long term drug use cause organ failure?”
- “Does insulin cause harmful effects to the body?”
- “Do you think that use of Thebu leaves (*Costus speciosus*) or Karivila (*Momordica dioica*) is better for diabetes as medicine than the tablets prescribed by doctors?”
- “Do you think that the alternative treatments (acupuncture, chiropractic treatments, yoga, hypnosis, Bali Thovil (a traditional ancient treatment modality in Sri Lanka), relaxation exercises or herbal remedies are better than usually prescribed methods (diet control and medications)?”
- “Do you believe that there is not much use in trying to have good blood sugar control, because complications of diabetes will happen anyway?”

Responses to above questions were assessed with categorical responses “Yes”, “No” and “Don’t know”. Participants who got four or more marks out of seven were categorized as having positive attitudes.

Practices were assessed using four questions on preventive strategies; self-care, dietary modifications and monitoring of blood sugar. Responses to the individual questions were assessed in order to evaluate the positive and negative practices.

### Ethical approval

Ethical approval for this study was obtained from ethical review committee of Faculty of Medicine, University of Ruhuna, Galle. Informed written consent was obtained from all individuals prior to data collection. Participants were informed of their rights to withdraw from the study at any stage.

### Statistical analysis

Participant’s socio-demographic characteristics including age, gender, level of education were reported using descriptive statistics. The knowledge on diabetes was compared between gender, age categories, and level of education using Chi-square tests. Statistical software SPSS (SPSS Inc., version 11) was used in the analysis of data.

## Results

Out of 277 of total participants, 58.5% were females and the mean age was 40 (±11.3) years. Majority had good education with 55% had studied up to advanced level (A/L) and only around 2% had education up to grade 5. More than one fourth of the study subjects were employed on fulltime basis and 27.4% were unemployed (Table 1). Interestingly, around 97% of study subjects have heard about diabetes previously.

**Table 1:** Socio-demographic characteristics of the study participants (*N* = 277)

Variable		Number	Percentage %
Age	18–34	113	40.8
	35–64	156	56.3
	Above or equal to 65	8	2.9
Gender	Male	115	41.5
	Female	162	58.5
Ethnic group	Sinhalese	269	97.1
	Sri Lankan Moors	8	2.9
	Tamil	0	0
Level of education	Up to grade 5	6	2.2
	Up to O/L^a^	49	17.7
	Up to A/L^b^	154	55.6
	Graduate	68	24.5
Employment status	Working fulltime	95	34.2
	Working part-time	25	9.0
	Unemployed	76	27.4
	House wife	42	15.1
	Retired	2	0.7
	Others^c^	37	13.3

^a^Ordinary level examination at grade 10

^b^Advanced level examination at grade 12

^c^Schooling, attending degree courses

### Knowledge assessment

Knowledge was measured using 8 main questions related to diagnosis, risk factors, prevention, and complications of DM. The mean (CI) knowledge score of the total sample was 16.5 (0.51). Around 37% of the participants scored 19 or more out of 26, and was categorised as having good level of knowledge. Out of the total score of 26, 23% of participants scored less than 14 (poor knowledge) and 39% scored between14 to 18 points (moderate knowledge). With regard to the pathophysiology of diabetes, 87% were aware that diabetes is a disease characterised by elevated blood sugar.

When asked, which organ dysfunction was associated with diabetes mellitus, 48% (*n* = 133) correctly mentioned it as the pancreas, 18% (*n* = 50) mentioned the liver, and 22% (*n* = 89) did not know the answer. In the multiple logistic regression analysis (Table 2), knowing pancreas as the primary organ involved in DM was significantly associated with younger age (age < 35 years) and tertiary education.

**Table 2:** Predictors of participant’s knowledge on the main organ involved in diabetes mellitus and its effect on brain

Variable	Primary organ dysfunction in DM- Pancreas^a^		DM affect brain as a complications^a^	
	p	OR (95% CI)	p	OR (95% CI)
Age < 35 years	0.01	0.67 (0.38–0.89)	0.09	1.09 (0.71–1.42)
Male gender	0.89	1.12 (0.83–1.34)	0.34	1.45 (0.56–2.12)
Tertiary level of education	0.02	1.74 (1.32–2.06)	0.16	1.02 (0.67–1.62)
Working fulltime	0.45	1.02 (0.63–1.38)	0.95	0.96 (0.45–1.56)

^a^Predictors of awareness on organ involved in diabetes mellitus through multivariate logistic regression

**Fig. 1:**
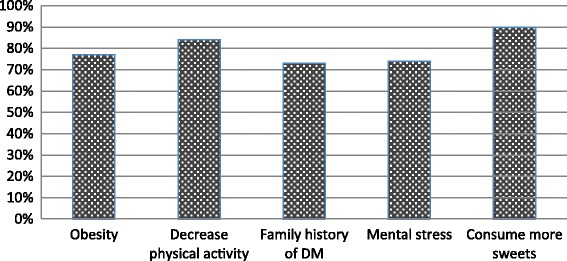
Participants knowledge on risk factors for diabetes mellitus

Regarding complications, 85% of the study subjects knew that diabetes could affect other organs. However, majority (56%) were unaware that DM can affect the brain (Table 3). Only the younger individuals (age < 35 years) had significantly better knowledge on the effect of DM on the brain (Table 2). Among the study subjects, 83% knew that diabetes can be prevented by appropriate measures. More than half of participants correctly answered to each of the questions on risk factors for DM. eg:- obesity leads to DM (77%), decrease physical activity may lead to DM (84%), having DM among family members is a risk factor for DM (73%) (Fig. 1). In addition, close to 90% were aware that consuming more sugar can lead to DM.

**Table 3:** Participants knowledge on effect of diabetes on other organs

Question	Yes (%)	No or Don’t know (%)
Can diabetes cause damage to the other organs of the body?	85	15
If yes, which of the following organs are affected in diabetes?		
Heart	50	50
Kidney	86	14
Brain	44	56

The association of knowledge with gender, level of education, and socio economic status were measured using chi square tests. There was no significant association between gender and age with knowledge on diabetes. However, level of education was significantly associated with knowledge (*p* = 0.001).

### Attitudes assessment

**Fig. 2:**
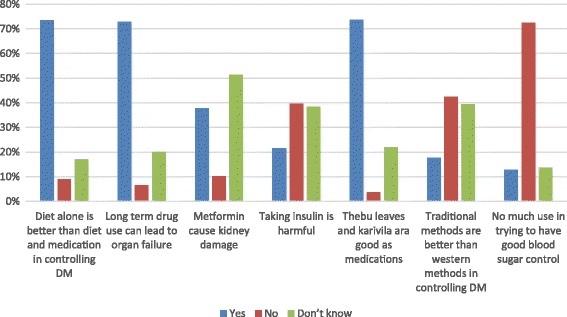
Individual responses to seven questions on attitudes

Attitude was assessed with seven questions and participants who got 4 or more marks were considered as having positive attitudes. Surprisingly, majority (88%) had poor attitude towards diabetes. About 73% believed that long term use of medications for diabetes will eventually lead to organ dysfunction. Close to 38% of participants who have heard about metformin believed that long term use of metformin can lead to kidney damage. Around 73% believed that use of alternative medicine such as Thebu leaves (*Costus speciosus*) was more beneficial than the standard treatments. Around 20% of participants thought that long-term use of daily insulin injections was harmful even when it was indicated to control blood sugar. Furthermore, about 18% believed that the other complementary and alternative treatments such as acupuncture, bali-thovil (traditional devil dance), herbal remedies, etc. were better in controlling DM than the usual methods such as diet and medications (Fig. 2). Unlike knowledge, attitude had no significant association with level of education. In addition, other factors such as knowledge on DM, gender, and socio-economic status were also not associated with attitudes.

### Practices assessment

**Fig. 3:**
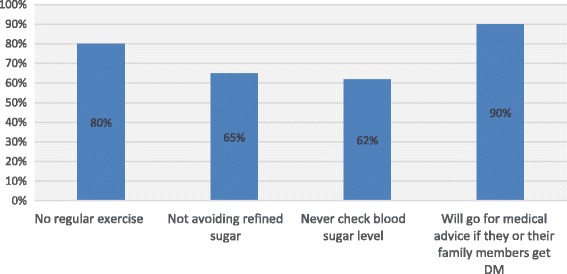
Diabetes preventive practices of participants

Practices were assessed using questions on participant’s intention to seek treatment, and preventive measures such as screening for DM, diet and, exercise. The majority (90%) stated that they will seek some form of treatment if they or their family members are found to have DM (Fig. 3). However, more than half of study subjects had never checked their blood sugar level and only around 30% had regular screening for DM with annual blood glucose measurements. About 65% take refine sugar liberally and a large majority (80%) didn’t involve with regular exercises (Fig. 3). After adjusting for the covariates, individuals with good socio-economic status had regular blood sugar measurement (Table 4) than people with poor socio economic status (*p* = 0.005).

**Table 4:** Associations of socio-demographic characteristics with regular blood glucose check

	OR^a^	95% CI	p
Age			
Less than 35 years	1.21	0.91, 1.48	0.19
35 or more	1.0		
Gender			
Male	1.08	0.92, 1.21	0.11
Female	1.0		
level of education			
Up to grade 5	1.0		
Up to O/L	1.05	0.82, 1.23	0.42
O/L or above	1.12	0.93, 1.31	0.32
Socio-economic			
Poor	1.0		
Good	1.3	1.07, 1.54	0.005

^a^Odd ratio (*OR*), 95% confidence interval (*CI*) adjusted for age, gender, level education and socio-economic status

## Discussion

This study is the first study from Southern Sri Lanka to report on knowledge, attitude and practice related to diabetes among general public. Noteworthy findings of this study are (1) majority had either moderate or good knowledge on diabetes, (2) there was no significant relationship between knowledge on diabetes with gender or age, (3) not surprisingly, level of education was significantly associated with knowledge on diabetes, (4) even though the knowledge was satisfactory in the majority of participants, their attitude towards diabetes was very poor, (5) majority were keen to seek some form of treatment if they or their family members develop DM, and (6) majority of study participants were not involving with any preventive strategies against diabetes mellitus.

Unlike most studies from developing countries which reported poor knowledge of diabetes among general public, this study shows that the levels of knowledge is comparatively better, with the majority (>75%) having either moderate or good knowledge [8, 10, 15–17]. Even though, the different studies used different instruments and/or were carried out among different ethnic or age groups, it is still a notable finding in this study. Even though we didn’t assess how individuals acquired the knowledge on diabetes, it is possible that higher literacy rate as well as well-developed social and media networks in Sri Lanka may have positive effect on their knowledge towards diabetes. Our study also revealed that the gender or age had no significant association with knowledge on diabetes. This finding also different to most other studies conducted in developing countries [8, 12, 16] where males were found to have better knowledge on the disease than females. Similar to many other studies, our study too showed a significant association between levels of education with knowledge [7, 12, 16, 18].

The most notable finding in our study is the gap between knowledge on diabetes and attitudes towards diabetes and its managements. Even though the majority (>75%) had either moderate or good knowledge it is not reflected on their attitudes as about 88% were found to have poor attitude towards diabetes. Many previous studies in similar setting to Sri Lanka have reported poor attitudes, but these studies reported poor knowledge too [8, 10, 16, 18]. Most studies show that the attitude goes in hand in hand with the knowledge. Even though it is difficult to find out the reasons for the gap between these two domains with a quantitative research like this, there may be number of plausible reasons. One reason may be improper and uncoordinated health education. In Sri Lanka, most of the diabetes health promotion efforts are conducted in uncoordinated and ineffective manner by different stakeholders especially in media networks. This might lead to false beliefs among general public. For an example, in our study, about 73% believed that long term use of medications such as metformin will lead to organ damage or dysfunction. Furthermore, about 73% thought Thebu leaves (*Costus speciosus*) were superior to standard anti-diabetic medications. Our study showed that almost 90% of general public had some idea about diabetes, but what they knew had no positive effect on attitude towards diabetes. There is evidence that poor attitude causes deleterious effect on diabetes management. In one of the study conducted among diabetic patients followed up in a tertiary care centre in Sri Lanka had revealed around 75% use some form of herbal medicine with or without anti-diabetic medications [19]. It is therefore clear that some of these beliefs directly influence the management of diabetes. Alarmingly, significant proportion of study subjects believed that metformin causes renal damage (37.8%) and taking insulin was harmful (21.5%). These wrong beliefs too can directly influence the diabetes management in our society. Therefore, we believe that it is necessary to direct more resources to improve the knowledge and develop an innovative educational model to change the attitude of general public. Knowledge does not always result in good attitude or positive behavioural changes. A previous study that examined the effect of knowledge on behaviour showed that the participants continued to take sweetened foods even though they were well aware about the deleterious effects of sugar on oral hygiene [20]. It is therefore important to identify interventions that reinforce peoples’ attitudes despite their levels of knowledge. Further in-depth research on diabetic patients’ knowledge, attitudes and practices and how they interact is a prerequisite for such effort.

Our study also shows that there is significant gap between knowledge and practices. Over 50% study population did not involve with any preventive measures against diabetes including restricting refine sugar (65%), regular exercises (80%) or checking blood sugar at least on in frequent basis (58%).

### Limitations of the study

Participants for this study were recruited at the household level by an open invitation method and hence can introduce a health-seeking bias with more health conscious people being inadvertently included in the study. Therefore, the results may not be truly representative of general public. However, this raises concerns that people who are less health conscious may have even poorer knowledge, attitudes and practices towards diabetes than what were observed in this study. Furthermore, we did not ask the sources of health information. Knowledge of the sources of information would have been useful in identifying the most appropriate method for health promotion among general public in Sri Lanka.

## Conclusion

This study provides a snapshot of the current situation of knowledge, attitude and practice related to diabetes in general population in Southern Sri Lanka. Even though the majority had adequate knowledge on diabetes, there is still some room for improvement as one fourth of study participants had poor knowledge on various aspects of diabetes. The most interesting finding of this study was the gap between knowledge and attitude towards diabetes and its management. Even though the majority had above average knowledge it was not reflected on their attitudes towards DM. Therefore, this study can be used as a baseline for the national diabetes awareness campaigns and modify the approach towards education on diabetes giving more emphasis on attitude change.

## Abbreviations

DM: Diabetes mellitus
KAP: Knowledge, attitude and practice
MOH: Medical Officer of Health
